# A polygenic score for executive function correlates with differential rates of cognitive decline

**DOI:** 10.1002/alz.094587

**Published:** 2025-01-09

**Authors:** Nadia Dehghani, Lauren Massimo, Emma Rhodes, Katheryn A Q Cousins, Rory Boyle, Sheina Emrani, Jeffrey S Phillips, Daniel T Ohm, Melanie A Matyi, Vivianna M Van Deerlin, David J Irwin, Corey T McMillan

**Affiliations:** ^1^ Penn Frontotemporal Degeneration Center, Department of Neurology, Perelman School of Medicine, University of Pennsylvania, Philadelphia, PA USA; ^2^ Center for Neurodegenerative Disease Research, Department of Pathology and Laboratory Medicine, Perelman School of Medicine, University of Pennsylvania, Philadelphia, PA USA

## Abstract

**Background:**

Executive dysfunction is a hallmark clinical feature of frontotemporal degeneration (FTD). Genome‐wide association studies have identified genetic variants, and resulting polygenic scores (PGS), related to executive function (EF) in population studies. We evaluated whether EF‐PGS correlates with differential rates of cognitive decline in FTD.

**Method:**

We computed an EF‐PGS based on a prior EF‐GWAS for 375 individuals genotyped in the Penn Integrated Neurodegenerative Disease Database with a diagnosis consistent with FTD including bvFTD, naPPA, svPPA or cognitively normal. We investigated the association between EF‐PGS tertiles and cognitive decline over time (letter‐guided fluency, n = 257; semantic fluency, n = 185 with animals, n = 171 with vegetables; backwards digit span, n = 140; Boston Naming Test, n = 179) in linear mixed effects models adjusting for age at baseline, sex, education years and baseline cognitive score.

**Result:**

Overall, the low‐PGS tertile exhibited faster decline over time in letter‐guided fluency assessed by the number of correct words beginning with the letter “F” produced in 1‐minute (t = −2.77, df = 96, *p* = 0.007 compared to the intermediate‐PGS tertile; t = −2.41, df = 99, *p* = 0.018 compared to the high‐PGS tertile). There was no significant differential rate of decline between EF‐PGS tertiles in measures of semantic fluency, confrontation naming (Boston Naming Test) or working memory (backwards digit span), with the caveat that fewer participants in our cohort had these measures.

**Conclusion:**

Our results suggest that lower EF‐PGS correlates with faster cognitive decline over time, particularly specific to letter‐guided fluency. This PGS, based on genetic variants associated with EF, may reflect the domain‐specific decline in cognitive performance observed across FTD syndromes.

